# Engineering optical mode ferromagnetic resonance in FeCoB films with ultrathin Ru insertion

**DOI:** 10.1038/srep33349

**Published:** 2016-09-15

**Authors:** Shandong Li, Cuiling Wang, Xian-Ming Chu, Guo-Xing Miao, Qian Xue, Wenqin Zou, Meimei Liu, Jie Xu, Qiang Li, Youyong Dai, Shishen Yan, Shishou Kang, Yunze Long, Yueguang Lü

**Affiliations:** 1 College of Physics, Laboratory of Fiber Materials and Modern Textile, the Growing Base for State Key Laboratory, and Key Laboratory of Photonics Materials and Technology in Universities of Shandong, Qingdao University, Qingdao 266071, China; 2 Department of Cardiology, the Affiliated Hospital of Qingdao University, Qingdao 266100, China; 3 Institute for Quantum Computing, Department of Electrical and Computer Engineering, University of Waterloo, Waterloo, N2L 3G1, Canada; 4 National Laboratory of Solid State Microstructures, Nanjing University, Nanjing 210093, China; 5 Fujian Institute of Research on the Structure of Matter, Chinese Academy of Sciences, Fujian, 350002, China; 6 School of Physics, Shandong University, Jinan 250100, China; 7 Department of Physics, School of Science, Harbin Institute of Technology, Harbin, 150001, China

## Abstract

Ferromagnetic resonance (FMR) in soft magnetic films (SMFs) to a large extent determines the maximum working frequency of magnetic devices. The FMR frequency (*f*_r_) in an optical mode is usually much higher than that in the corresponding acoustic mode for exchange coupled ferromagnet/nonmagnet/ferromagnet (FM/NM/FM) trilayers. In this study, we prepared a 50 nm FeCoB film with uniaxial magnetic anisotropy (UMA), showing a high acoustic mode *f*_r_ of 4.17 GHz. When an ultrathin Ru spacer was inserted in the very middle of the UMA-FeCoB film, the zero-field FMR was abruptly switched from an acoustic mode to an optical one with *f*_r_ dramatically enhanced from 4.17 GHz to 11.32 GHz. Furthermore, the FMR mode can be readily tuned to optical mode only, acoustic mode only, or double mode by simply varying the applied filed, which provides a flexible way to design multi-band microwave devices.

Ferromagnetic resonance is one of the most fundamental physical phenomena of SMFs, which practically decides the operation speed of these magnetic materials in actual devices^1^,^2^. FMR-based radio-frequency (RF) and microwave devices are widely used in communication, information, aerospace, aviation, military, and many other fields, and higher and higher *f*_r_ is in demand with the fast advance of science and technology^3^,^4^. The coercivity (H_C_) of SMFs is expected to decrease with the grain size (D) following a relationship of 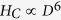, as a result of the random anisotropy mechanism^5^. Therefore, nanocrystalline SMFs, such as FeCo-based films, are promising in RF/microwave integrated circuit (IC) devices due to their strong saturation magnetization (4πM_S_), high *f*_r_, large permeability (μ), and good compatibility with IC processes. In recent years, with many researchers’ dedicated efforts to enhance the anisotropic field H_K_, the zero-bias *f*_r_ of FeCo-based SMFs was enhanced from several hundred MHz^6^,^7^ up to S-band (2–4 GHz)^8^,^9^,^10^ and C-band (4–8 GHz)^11^,^12^. Very recently, *f*_r_ has reached the range of X-band (8–12 GHz)^13^,^14^,^15^. However, it becomes increasingly difficult to drive FMR to higher frequencies solely from increasing H_K_. It is therefore highly desired to explore new ways of further increasing the FMR frequency.

FMR is known to show both acoustic and optical modes in exchange coupled FM/NM/FM trilayers. The interlayer exchange coupling (IEC) does not produce any dynamic contribution to the acoustic mode resonance because the RF components of the two magnetization vectors are tightly phase locked. Therefore, the acoustic mode dispersion relation in the trilayer is no different from that in a single-layer system. On the other hand, the IEC is able to introduce an extra effective field J_eff_ into the dispersion relation of the optical mode, in which the magnetization vectors resonate out-of-phase^16^. Therefore, the FMR frequency of the optical mode (*f*_r_^O^) will be much higher than that of the acoustic mode (*f*_r_^A^) in an antiferromagnetically (AFM) coupled trilayer. Moreover, in some trilayers, such as Fe/Cr/Fe, Co/Ru/Co, the FM layers are strongly AFM coupled by IEC field with the strength on the order of several kOe^17^,^18^, which can lead to a very high *f*_r_ up to 50 GHz. Nevertheless, applicable optical mode resonance in AFM coupled trilayers has not been reported. In general, the optical mode resonance in FM_1_/NM/FM_2_ trilayers is only observable in FMR measurements when the individual FM layers have different resonance fields or magnetizations^16^,^19^. The acoustic mode of FMR has been well utilized in practical RF/microwave devices, while the optical mode resonance is largely neglected due to its weak permeability^16^,^19^,^20^ until very recently, when we discovered good permeability and optical mode frequency in IEC trilayers^21^. In this article, we demonstrate well-engineered tunability on the microwave performance of Fe_0.5_Co_0.5_-B/Ru/Fe_0.5_Co_0.5_-B (abbreviated to FeCoB/Ru/FeCoB below) trilayers. It is exciting that controllable optical mode resonance with enhanced frequency (11.32 GHz) and high permeability (over 200 at resonance) was achieved in UMA-FeCoB films with ultrathin Ru spacer (ca. 4 Å). This work provides a new way of engineering SMFs towards higher *f*_r_ via optical mode instead of acoustic mode, for microwave materials and devices.

Self-biased SMFs used in RF/microwave ICs should have two important properties: uniaxial magnetic anisotropy, and nanocrystalline structure^5^,^6^,^7^,^8^,^9^,^10^,^11^,^12^,^13^,^14^. The former provides an effective magnetic field H_K_, leading to strong FMR in absence of external magnetic fields^6^,^7^,^8^,^9^,^10^,^11^,^12^,^13^,^14^. The latter will soften the magnetic properties leading to small coercivity and low magnetic loss^5^. Therefore, investigations on microwave SMFs so far mainly focus on the enhancement of H_K_ in nanocrystalline films. Several useful approaches, such as oblique sputtering^12^,^22^, exchange coupling^23^,^24^,^25^,^26^, and magnetoelectric coupling^27^,^28^, were developed for achieving higher H_K_. In our previous work^11^,^29^,^30^, a novel composition gradient sputtering (CGS) method was invented, where magnetic main target (e.g. Fe_0.5_Co_0.5_) has a normal incidence to the wafers, while doping element target (e.g. B) has an angled incidence to create the chemical gradient. A dramatically increase of the in-plane H_K_ up to 547 Oe is achieved due to the uniaxially distributed chemical stress induced by doping composition gradient. As a result, good microwave ferromagnetic properties with *f*_r_ over 7 GHz were obtained in CGS FeCoHf magnetic films^11^.

## Results

### The composition distribution of FeCoB single layer prepared by CGS method

The composition distribution along the radial (R) direction of sample turntable was confirmed by a field-emission electron probe microanalyzer (FE-EPMA). The atomic ratio of Fe:Co remains at 0.91 throughout the wafer, indicating a homogeneous composition comparable to the Fe_0.5_Co_0.5_ target composition due to its normal incidence; while the atomic ratio of B:Co increases linearly across the wafer, from 0.83 to 0.87 for the test distance ***x*** from 1 to 5 mm. It is the B composition gradient that results in the strong uniaxial magnetic anisotropy and good microwave ferromagnetic performance in these CGS-FeCoB films.

### Microstructure analysis

**Figure 1:**
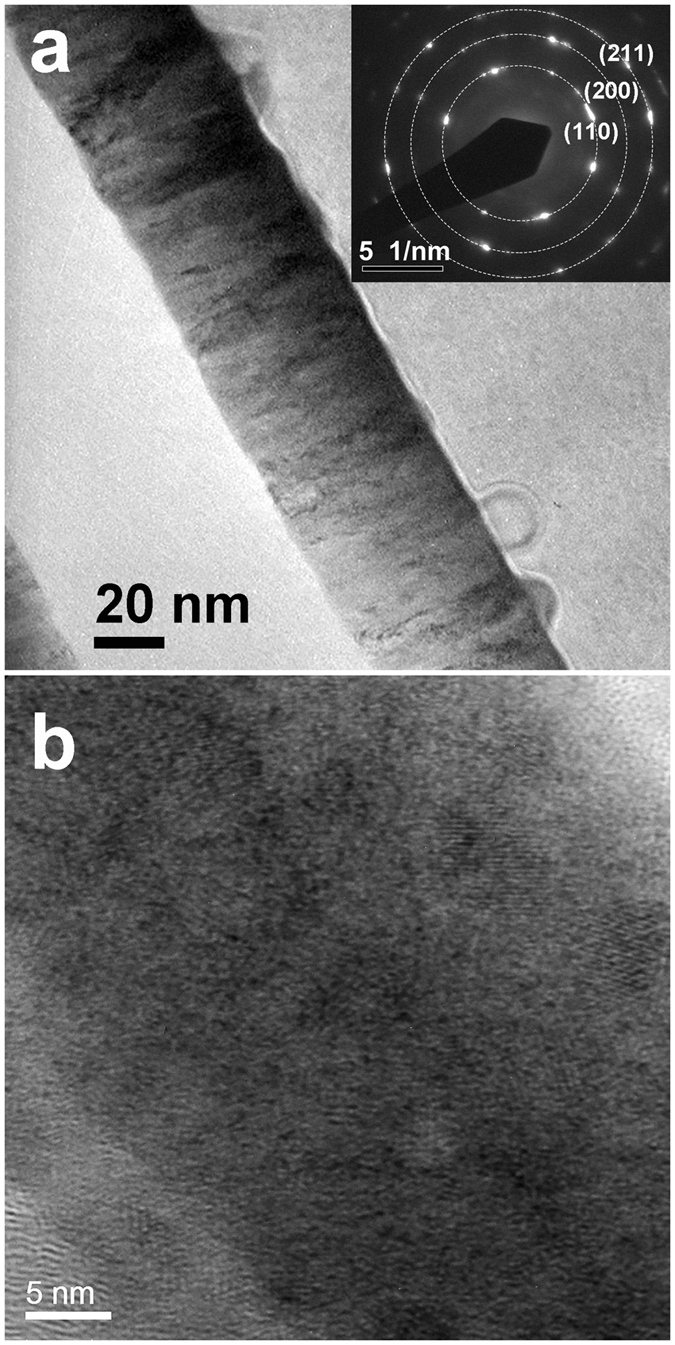
The morphology (**a**) and high-resolution image (**b**) of an UMA FeCoB single layer. The inset of (**a**) shows the electron diffraction spectroscopy pattern over a few nanocrystals and the corresponding facet indexes, where the dashed circles are guide for the eyes. (**b**) shows the nanocrystalline FeCoB with the average grain size smaller than 8 nm.

The microstructure of the FeCoB single layer was examined by a high-resolution scanning transmission electron microscope (HR-STEM). As shown in Fig. 1a, thickness of the FeCoB single layer is indeed 50 nm. The electron diffraction spectroscopy (EDS) pattern reveals that the FeCoB single layer has a polycrystalline bcc structure. The high-resolution image, shown in Fig. 1b, demonstrated that the average grain size of the FeCoB film is smaller than 8 nm, which is beneficial to the enhancement of microwave ferromagnetic performance.

### Uniaxial magnetic anisotropy

**Figure 2:**
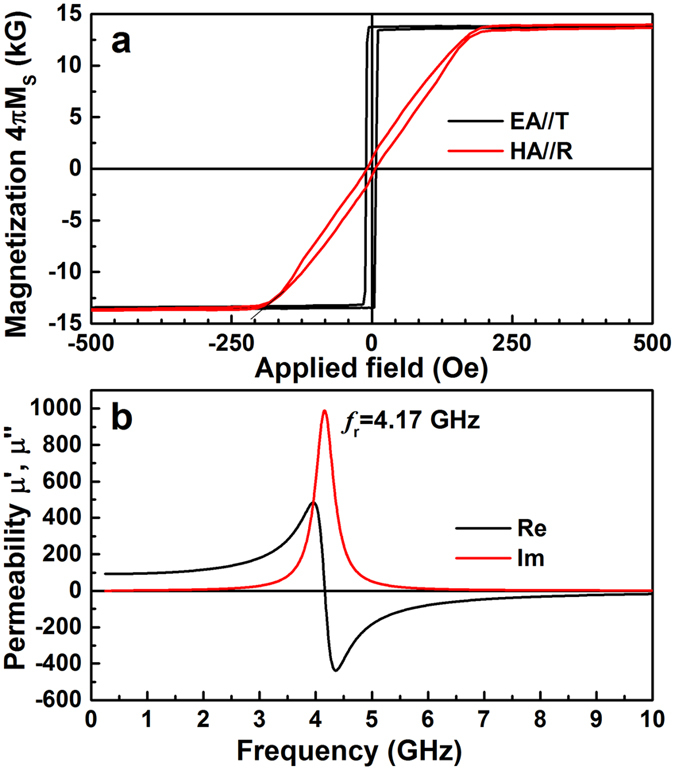
Hysteresis loops (**a**) and permeability spectra (**b**) for the UMA FeCoB single layer. A well-defined uniaxial magnetic anisotropy with easy and hard axes along tangential and radial direction of the sample turntable respectively, is present in CGS-FeCoB single layer (**a**). A sharp FMR peak at 4.17 GHz was observed (**b**).

Figure 2a demonstrates that the FeCoB single layer exhibits a well-defined uniaxial magnetic anisotropy with a large anisotropy field H_K_ of 195 Oe and its easy axis (EA) parallel to the tangential (T) direction of the sample turntable. Similar to the discussions in our previous work^11^,^29^,^30^, the gradient distribution of B gives rise to a gradient chemical stress, which then induces a large H_K_. According to the Kittel’s equation, 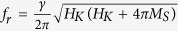, the large H_K_ leads to a high self-biased *f*_r_. Figure 2b showed that a high *f*_r_ of 4.17 GHz was achieved in the CGS-FeCoB single layer. In addition, the small grain sizes give rise to the desired low H_C_ of 8.8 Oe and good soft magnetic performance.

### The Ru thickness dependence of FMR performances for FeCoB/Ru/FeCoB trilayers

**Figure 3:**
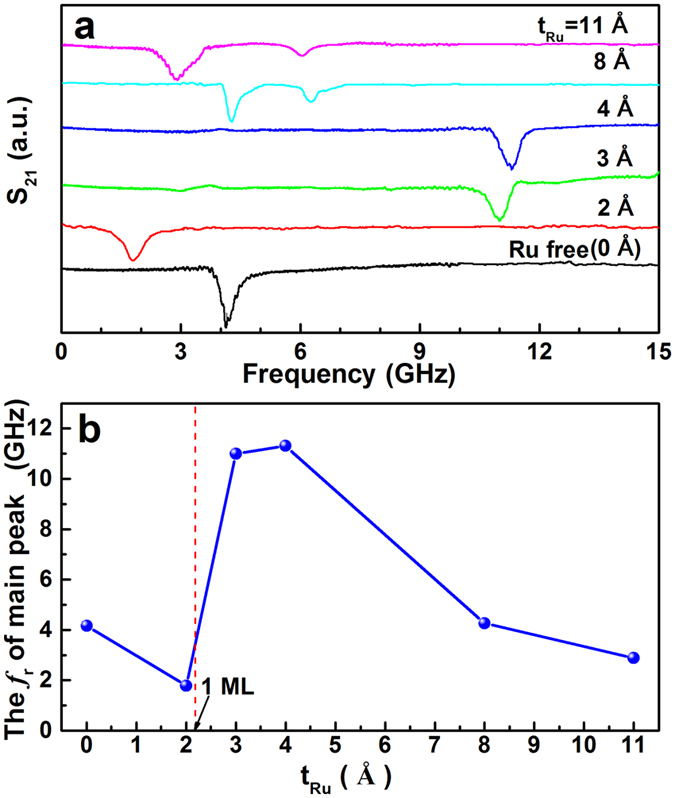
The Ru spacer thickness dependence of FMR frequency, (**a**) scattering coefficient S_21_ at zero magnetic field, and (**b**) the FMR frequency of the strongest FMR peak on each sample. The absorption peak of S_21_ represents the FMR frequency. For the sample without Ru spacer or with thinner ones, the FMR shows acoustic mode resonance with low frequency (t_Ru_ = 0 and 2 Å). When the Ru spacer is 3–4 Å (slightly thicker than 1 ML), only optical mode resonance is observed due to the extinction of the acoustic mode resonance (t_Ru_ = 3 and 4 Å). With further increasing the Ru thickness (t_Ru_ = 8 and 11 Å), the strength of interlayer exchange coupling is weakened and both optical and acoustic mode resonances co-exist.

Figure 3a shows the Ru thickness dependence of the scattering coefficient S_21_ at zero applied magnetic field. The S_21_ scattering parameter represents microwave transmission, and strong absorption (dip of the transmitted signal) happens around the FMR frequency. As illustrated, the CGS-FeCoB single layer (Ru free) shows a high *f*_r_ of 4.17 GHz, indicating a high UMA field H_K_ induced by the B composition gradient in the CGS film. When an ultrathin Ru spacer is inserted to the very middle of the 50 nm FeCoB film, the S_21_ spectra depend very sensitively on the inserted layer thickness. For the trilayer with a 2 Å Ru spacer, the *f*_r_ is reduced from 4.17 (single layer) to 1.82 GHz; however, for the trilayers with Ru thickness in the range of 3–4 Å, the *f*_r_ dramatically increases to more than 11 GHz. With further increasing the Ru thickness to 8 and 11 Å, the *f*_r_ is reduced again, but an additional absorption peak appears at lower frequencies. The FMR frequencies of the strongest absorption peaks are summarized in Fig. 3b. It is obvious that the highest *f*_r_ is present in trilayers with 3–4 Å Ru spacer, which is slightly thicker than 1 ML of Ru (a ML of hcp Ru is 2.14 Å if (001) orientated, and 2.34 Å if (110) oriented).

## Discussions

### The interface microstructure

**Figure 4:**
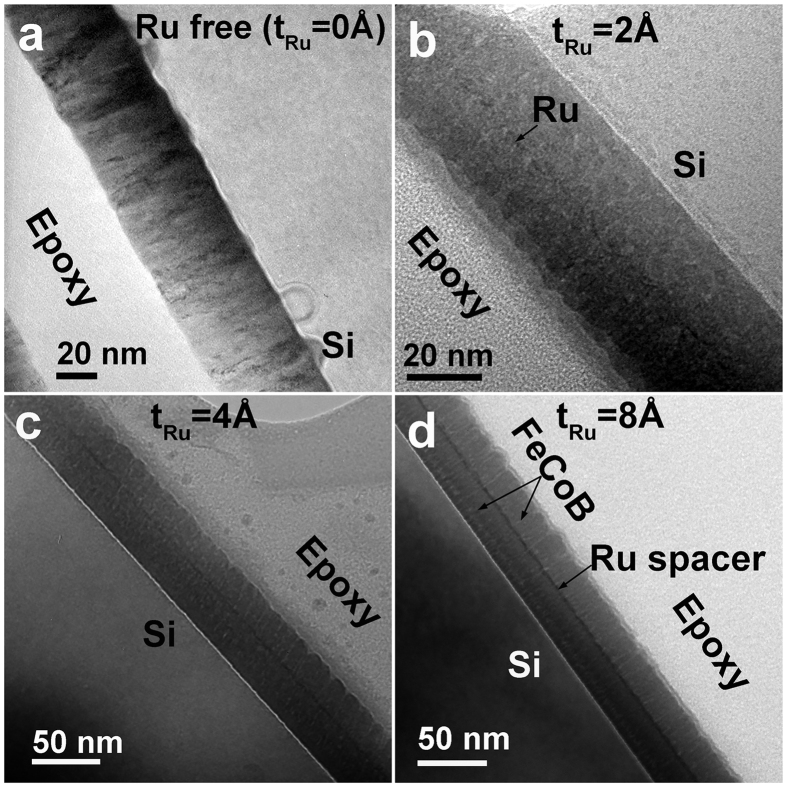
The cross-sectional TEM images of samples with different Ru spacer thicknesses, (**a**) FeCoB single layer, (**b**–**d**) FeCoB/Ru/FeCoB trilayers with t_Ru_ from 2 to 8 Å, respectively. With the increase of Ru thickness, the Ru spacer changes from discontinuous (**b**) to continuous (**c**,**d**).

In order to find out the reason that *f*_r_ is dramatically enhanced with inserting an ultrathin Ru, we carried out HR-STEM to check the microstructures of various samples. Figure 4 shows the comparison of cross-sectional TEM images for typical samples with Ru thickness of 0, 2, 4 and 8 Å, respectively. It should be mentioned that we cannot distinguish the Ru atoms in the middle from FeCoB nanocrystllines under the HR-STEM due to the Ru spacer roughness. Fortunately, the atomic radius of Ru (1.89 Å) is larger than that of Fe (1.72 Å) and Co (1.67 Å). The difference of atomic radius between Ru and Fe/Co results in a stress around the interface, which changes the trajectory of diffraction electrons and produces a stress-induced diffraction contrast^31^, indirectly revealing the interface information near the Ru spacer. As illustrated in Fig. 4b–d, the Ru spacer is still discontinuous in the trilayer with 2 Å Ru, while it becomes continuous for Ru thicker than 1 ML (e.g. 4 and 8 Å, Fig. 4c,d). As clearly revealed below, the interface varying from discontinuous to continuous results in an abrupt change of the FMR mode type and microwave properties.

### The effect of Ru spacer thickness on the static and alternative magnetic properties

**Figure 5:**
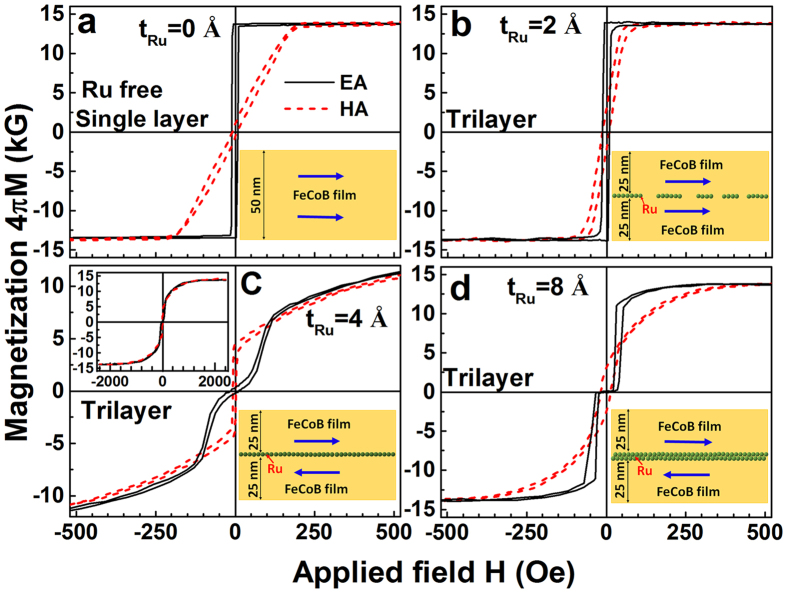
Hysteresis loops along EA and HA direction. The inset in each panel shows the schematic drawing of Ru atomic layer structure and the magnetic moment orientation in the corresponding sample. A well-defined uniaxial magnetic anisotropy is present in the UMA-FeCoB single layer (**a**). The trilayers’ hysteresis loops (**b–d**) are sensitive to the Ru thickness. For the trilayer with Ru thickness t_Ru_ = 2 Å (**b**), hysteresis loops similar to the single layer are observed with slightly larger H_C_ and smaller H_K_. For the trilayer with 4 Å Ru (**c**), a double S shape loop along EA direction is present. The upper left inset of (**c**) shows the hysteresis loop of the trilayer with 4 Å Ru under a wider magnetic field range, indicating that it does not fully saturate even at magnetic fields of 2 kOe. The trilayer with 8 Å Ru (**d**) shows a lower flip field, implying a weakened interlayer exchange coupling.

The hysteresis loops of the representative samples are summarized in Fig. 5. The insets in Fig. 5 show the schematic structure of the Ru layer and magnetic moment orientations of the FeCoB layers, which are deduced from the TEM images in Fig. 4 and hysteresis loops in Fig. 5. Comparing to the single layer (Fig. 5a), the trilayer with 2 Å Ru (less than 1 ML) shows a lower H_K_ and higher H_C_ (Fig. 5b), implying its microwave soft magnetic properties are deteriorated by the discontinuous Ru spacer. In this sample, the FM layers on the two sides of the Ru spacer are overall FM coupled since they are not entirely separated. The added AFM (Ru covered region) coupling weakens H_K_ and results in a lower *f*_r_ of 1.82 GHz in the trilayer with 2 Å Ru (Fig. 3).

For a Ru spacer thicker than 1 ML (e.g. 4 Å, Fig. 5c), the two FM sublayers on the two sides of the Ru spacer no longer have direct contact. The FM sublayers change abruptly from ferromagnetic coupling to antiferromagnetic coupling, and the hysteresis loops are far different from those of the single layer (Fig. 5a,c). Its EA hysteresis loop shows a double S shape with near-zero remanence. This is a clear demonstration of spin-flop switching, where the system goes abruptly from an AFM-like configuration to a FM-like configuration^32^,^33^. One can already visualize that an AFM configuration suppresses the in-phase acoustic resonance modes while a FM configuration suppresses the out-of-phase optical resonance modes. The forward and reverse magnetization curves are very close to each other, but do not completely overlap until reaching very large magnetic fields. As shown in the inset of Fig. 5c, the hysteresis loops do not saturate even at an applied field of 2 kOe. These features indicate that there exists strong antiferromagnetic interlayer coupling between the neighboring FeCoB sublayers through the ultrathin Ru layer^34^. As discussed above, the strong antiferromagnetic IEC in the trilayer with a 4 Å Ru spacer results in an optical mode resonance with very high *f*_r_. Therefore, the sudden jump of *f*_r_ from 4.17 GHz in the single layer to 11.32 GHz in the trilayer with a 4 Å Ru spacer can be attributed to the resonance mode varying from an acoustic mode in the single layer to an optical mode in the trilayer. In other words, inserting an ultrathin Ru spacer (>1 ML) in the middle of UMA-FeCoB film results in two separated FM sublayers coupled with AFM IEC, leading to qualitative changes of magnetic coupling type, FMR mode, and magnetic properties, and ultimately the pronounced difference of microwave performances between the single layer and the trilayer.

When the two FM sublayers are strictly antiparallel to each other, the OM resonance, even though still exists, has essentially zero net magnetization thus no observable resonance amplitude. Further increasing the Ru thickness (e.g. 8 Å, Fig. 5d), the strength of IEC is reduced, and the moments in the two FM sublayers are no longer tightly coupled. Although the upper and lower FM sublayers are still AFM-like coupled, their moments can deviate from the strict antiparallel alignment. In this case, both parallel and antiparallel moment alignments between the two FM sublayers co-exist, leading to the observable co-existence of acoustic and optical mode resonances (Fig. 3). As the coupling weakens, both the AM and OM frequencies do approach the single layer AM frequency, but not monotonically due to the oscillating RKKY coupling strength.

### The magnetic field dependence of FMR modes in the single layer and trilayers

**Figure 6:**
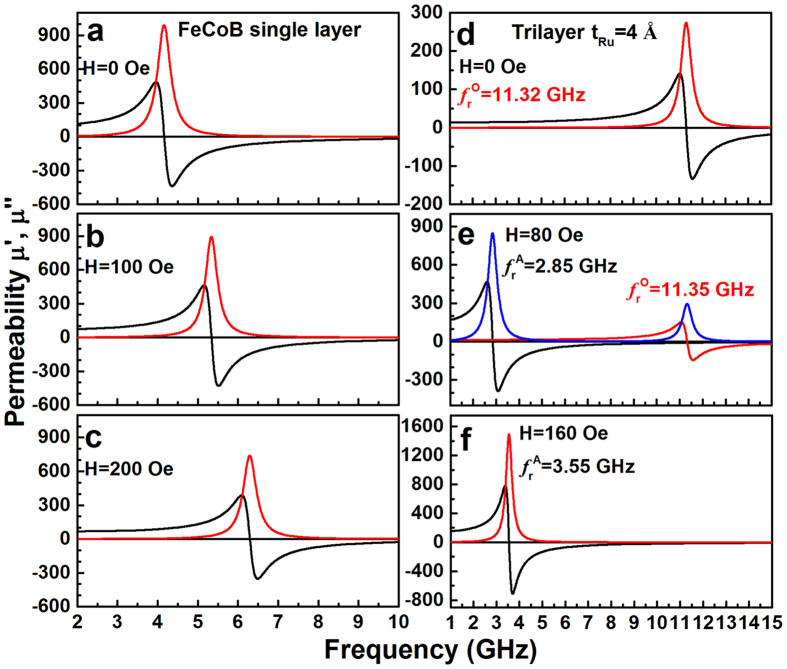
The magnetic field dependence of permeability spectra along EA direction. The FMR frequency peak in FeCoB single layer increases monotonically with the increase of magnetic field due to its acoustic mode FMR (**a–c**). However, the FeCoB/Ru/FeCoB trilayer exhibits three possible FMR mode: optical only (**d**), acoustic only (**f**), or double mode (**e**), depending on the magnetic configuration at the corresponding external magnetic field.

Unlike the acoustic mode, the FMR optical mode is sensitive to the relative magnetic moment orientation in the two FM sublayers. Therefore, these two modes can be distinguished by changing the applied field. Figure 6 shows the permeability spectra at various magnetic fields for the single layer and the trilayer with 4 Å Ru spacer. As illustrated in Fig. 6a–c, the single layer exhibits an acoustic mode resonance and its *f*_r_ monotonically increases with the applied field. However, the magnetic field dependence of permeability spectra for the trilayer with 4 Å Ru spacer is quite different from that of the single layer, showing some interesting and complex characteristics. An ultrahigh *f*_r_ of 11.32 GHz is achieved in the trilayer at zero applied magnetic field, which can be readily assigned to the optical mode resonance (Fig. 6d). Under an applied field of 80 Oe, besides a high FMR peak at 11.35 GHz (optical mode resonance), an additional resonance peak at 2.85 GHz (acoustic mode resonance) appears (Fig. 6e). Further increasing the applied field to 160 Oe, the FMR peak at high frequency disappears and only one FMR peak at 3.55 GHz (acoustic mode) is observed (Fig. 6f).

In general, the optical mode resonance is inaccessible in isotropic systems^16^. However, a strong optical mode resonance with high frequency was achieved in this study. It can be attributed to the engineered difference in magnetic properties between the studied UMA-FeCoB trilayers and the conventional trilayers: (1) the moments in the latter are orientated randomly, while those in the former are self-aligned along the EA direction (transverse to the composition gradient direction) due to the uniaxial magnetic anisotropy induced by chemical stress; (2) the large UMA field H_K_ makes the moments precess around the direction of H_K_ even at zero applied field; (3) the ultrathin Ru spacer gives rise to a strong AFM interlayer coupling, leading to a dramatic enhancement of the optical mode resonance frequency. The combination of these factors results in an enhancement of optical modes and suppression of acoustic modes at zero applied field.

**Figure 7:**
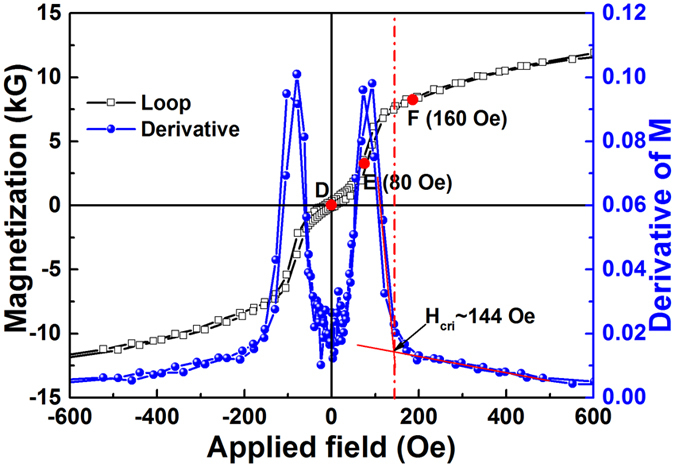
The hysteresis loop along EA and its first derivative for the trilayer with a 4 Å Ru spacer. Some representative points of D (zero field), E (magnetic moments fast flipping) and F (approaching saturation) are marked in Fig. 7. The off-set magnetic field of 144 Oe in the derivative curve refers to a critical field H_cri_, where most of magnetic moments antiparallel to the external field are flipped to the direction of applied field. (Points D, E, F are where Fig. 6d–f are measured).

In order to clarify the relationship between the FMR modes and the applied field, the hysteresis loops of the single layer and the trilayer with a 4 Å Ru spacer are carefully compared. As shown in Fig. 5a, the hysteresis loop of the single layer along EA shows a very small coercivity of 8.8 Oe and very large remanence ratio close to 1, indicating that the magnetic moments of the single layer are arranged along the EA direction. When the applied field is also along the EA direction, the moments will precess uniformly, forming an acoustic mode resonance. The FMR frequency will increase monotonically with the applied field (Fig. 5a–c). However, the trilayer with an ultrathin Ru spacer shows a complex magnetization process. For the studied trilayer, the equilibrium state is governed by the competition among three types of energies: (1) the uniaxial magnetic anisotropy energy induced by B composition gradient, which pushes the moments towards the uniaxial EA directions; (2) the interlayer exchange coupling energy due to the separation by an ultrathin Ru spacer, which prefers to align the moments of the two FeCoB sublayers antiparallel; (3) the Zeeman energy caused by the applied field, which drives the moments towards the applied field direction. Figure 7 shows the hysteresis loop of the trilayer with a 4 Å Ru spacer along EA and its first derivative, where the representative locations for measuring the permeability spectra in Fig. 6d–f are marked using D, E(80 Oe) and F(160 Oe), respectively. Site D refers to the location of zero-bias magnetic field, E locates at the site where the moments flip towards the applied field fast, and F refers to the field where the magnetization is approaching saturation. From the first derivative curve, it can be seen that a critical field H_cri_ is present due to the competition among the three types of energies, where the interlayer AFM coupling is overcome by the Zeeman energy and the two FM sublayers change from antiferromagnetic-like to ferromagnetic-like alignment. H_cri_ of 144 Oe is deduced from the off-set field of the first derivative curve, as shown in Fig. 7. The three permeability spectra measurement points are distributed on both sides of H_cri_ (E and F) and at the origin of the coordinates (D).

At site D with zero applied field, no Zeeman energy is present, the combination of the uniaxial magnetic anisotropy energy and the interlayer exchange coupling makes the moments of the two FM sublayers antiparallel to each other and orientated along opposite EA directions. Thus, only optical mode resonance with ultrahigh FMR frequency of 11.32 GHz was observed (Fig. 6d).

With the increase of magnetic field along the EA direction (from D to E site), the magnetization increases slowly in the beginning because the rotation of the moments in one of the FeCoB sublayers (the layer initially antiparallel to the applied field) is impeded by the anisotropy energy barrier, and a magnetization plateau shows up in the loop. On the other hand, the strong interlayer AFM coupling forces the moments that are initially parallel to EA to deviate from the EA direction. Therefore, at site E with an applied field of 80 Oe, the collinear antiferromagnetic moment alignment is distorted, leading to a non-collinear antiferromagnetic-like moment alignment with an obtuse angle between the sublayer moments. At this field, both parallel and antiparallel moment alignments coexist, therefore both acoustic and optical mode resonances are present simultaneously (Fig. 6e).

When the applied field increases to beyond the critical field H_cri_ (such as site F with an applied field of 160 Oe), the moments that are initially antiparallel to the applied field have already overcome the anisotropy energy barrier and flipped towards the applied field, forming a ferromagnetic-like moment alignment with an acute angle. At this stage, the acoustic mode dominates the permeability spectra, and the optical mode resonance is too small to be observed (Fig. 6f).

The UMA-FeCoB trilayer separated by an ultrathin Ru spacer (4 Å) exhibits intriguing microwave soft magnetic performances. It presents three types of resonance characters depending on the applied field: the high-frequency optical mode, the low-frequency acoustic mode, and the coexistent double mode. These features are especially suitable for applications in multiband microwave devices, which can work at high-, low-, or double-frequencies, with simply changing the applied fields. The excellent microwave soft magnetic performance of the trilayers can be attributed to their special magnetic structures. One remarkable feature of the CGS-FeCoB films, quite different from the conventional isotropic SMFs, is that their magnetic moments prefer to align with EA set by the chemical stress from the B composition gradient. This feature is very important because the orientated moments of the FeCoB sublayers ensure the observable optical mode resonance at zero applied field. Otherwise, the optical mode resonance will be averaged out as in randomly orientated films. The AFM-like moment alignment persists till a critical field H_cri_, beyond which the anisotropy field is overcome by the applied field and the moment alignment becomes more FM-like. As a result, three resonance regions with high, low and double FMR frequencies are present via controlling the applied field.

In conclusion, this work provides a new way to prepare soft magnetic films with higher *f*_r_ via optical mode, instead of acoustic mode as in conventional films. FeCoB nanocrystalline soft magnetic films with ultrathin Ru insertion and uniaxial magnetic anisotropy were prepared by a composition gradient sputtering method. The inserted Ru spacer layer results in an antiferromagnetic interlayer exchange coupling, which leads to an enhanced optical mode resonance with ultrahigh FMR frequency of 11.32 GHz at zero bias field. Moreover, such trilayers exhibit three distinct resonance performances: the high-frequency optical mode, the low-frequency acoustic mode, and the coexistent double mode, all of which can be readily tuned with simply varying the applied field. These features provide a flexible design for multiband microwave devices, which can function at high-, low-, or double-frequencies.

## Methods

### Sample preparation

A single crystal (100) Si substrate with the dimension of 5 × 5 × 0.5 mm^3^ was loaded on a turntable. The first UMA-FeCoB layer of 25 nm in thickness was deposited on the substrate by the CGS method at room temperature under 2.8 mTorr Ar pressure with a flow rate of 20 sccm, along with a RF power of 80 W on one Fe_0.5_Co_0.5_ target (normal incidence) and 120 W on one B target (angled incidence to create the chemical gradient). Then an ultrathin Ru layer was deposited on top of the first UMA-FeCoB layer. After that, the second 25 nm UMA-FeCoB layer was deposited under the same preparation conditions. In order to study the effect of Ru spacer thickness on the microwave ferromagnetic performances, various Ru thicknesses from 2 to 11 Å were deposited and a series of uniaxial magnetic anisotropic and antiferromagnetically coupled FeCoB/Ru/FeCoB trilayers were obtained. As a control sample, an UMA-FeCoB single layer with thickness of 50 nm was also deposited under the same conditions.

### Samples characterization

The thickness of the films is determined by deposition rate and time. The composition distribution was detected by a FE-EPMA with model JXA-8500 F. The magnetic properties and the microwave performance were measured by a physical property measurement system (MPMS, Quantum Design Co. Evercool II) and a vector network analyzer (Agilent N5224A) with a co-planar waveguide transmission line fixture. The vector network analyzer records the scattering parameters, and simulates the measured curves with the Landau-Liftshitz-Gilbert (LLG) equation. The microstructures observation of the films were carried out by a HR-STEM (FEI Co. Tecnai F20).

## Additional Information

**How to cite this article**: Li, S. *et al*. Engineering optical mode ferromagnetic resonance in FeCoB films with ultrathin Ru insertion. *Sci. Rep.* **6**, 33349; doi: 10.1038/srep33349 (2016).
